# Mapping competitive pathways to terpenoid biosynthesis in *Synechocystis* sp. PCC 6803 using an antisense RNA synthetic tool

**DOI:** 10.1186/s12934-023-02040-2

**Published:** 2023-02-23

**Authors:** João S. Rodrigues, Barbara Bourgade, Karen R. Galle, Pia Lindberg

**Affiliations:** 1 Department of Chemistry – Ångström, Uppsala University, Uppsala, Sweden; 2 Faculty of Sciences, University of Porto, Porto, Portugal

**Keywords:** Antisense RNA, Metabolic engineering, Terpenoids, Isoprene, Bisabolene, *Synechocystis* sp. PCC 6803

## Abstract

**Background:**

*Synechocystis* sp. PCC 6803 utilizes pyruvate and glyceraldehyde 3-phosphate via the methylerythritol 4-phosphate (MEP) pathway for the biosynthesis of terpenoids. Considering the deep connection of the MEP pathway to the central carbon metabolism, and the low carbon partitioning towards terpenoid biosynthesis, significant changes in the metabolic network are required to increase cyanobacterial production of terpenoids.

**Results:**

We used the Hfq-MicC antisense RNA regulatory tool, under control of the nickel-inducible P_*nrsB*_ promoter, to target 12 different genes involved in terpenoid biosynthesis, central carbon metabolism, amino acid biosynthesis and ATP production, and evaluated the changes in the performance of an isoprene-producing cyanobacterial strain. Six candidate targets showed a positive effect on isoprene production: three genes involved in terpenoid biosynthesis (*crtE*, *chlP *and *thiG*), two involved in amino acid biosynthesis (*ilvG* and *ccmA*) and one involved in sugar catabolism (*gpi*). The same strategy was applied to interfere with different parts of the terpenoid biosynthetic pathway in a bisabolene-producing strain. Increased bisabolene production was observed not only when interfering with chlorophyll *a* biosynthesis, but also with carotenogenesis.

**Conclusions:**

We demonstrated that the Hfq-MicC synthetic tool can be used to evaluate the effects of gene knockdown on heterologous terpenoid production, despite the need for further optimization of the technique. Possible targets for future engineering of *Synechocystis* aiming at improved terpenoid microbial production were identified.

**Supplementary Information:**

The online version contains supplementary material available at 10.1186/s12934-023-02040-2.

## Background

Terpenoids are a class of secondary metabolites that fulfil many different physiological roles in Nature. Besides their structural diversity, many of these compounds present chemical and physical properties relevant to Industry (e.g. as pigments, fragrances or feedstock for drugs and biofuel development) [1–3]. Photosynthetic microorganisms such as cyanobacteria are appealing host cells for microbial production of terpenoids, not only because they are able to produce terpenoids natively, but also because they do so using fixed CO_2_ and sunlight as carbon and energy sources.

Terpenoid biosynthesis in cyanobacteria starts from pyruvate and glyceraldehyde 3-phosphate (G3P), which are converted into isopentenyl pyrophosphate (IPP) and dimethylallyl pyrophosphate (DMAPP), the building blocks for all terpenoids, via the methylerythritol phosphate (MEP) pathway [4–7]. These two prenyl molecules are sequentially condensed, rendering terpene backbones with different chain lengths, which are then further modified to originate a wide range of terpenoids (e.g. carotenoids, squalene, phytol tail of chlorophyll *a*) [7].

Despite efforts in engineering cyanobacteria for enhanced terpenoid production [2, 8, 9], the rather low carbon partitioning towards this pathway constitutes a major issue. Only 5% of the carbon fixed by the cells is committed to terpenoids, and even with the introduction of heterologous pathways, most of the committed carbon still needs to be utilized for the biosynthesis of carotenoids, phytol and quinone prenyl tails [10]. Significant modifications in the core cyanobacterial metabolism are thus required to increase the carbon availability for terpenoids, especially if biotechnological applications are considered. The elimination of competing pathways may pose challenges, considering that the substrates used for the MEP pathway are deeply connected to the central carbon metabolism and inactivating such pathways may cause detrimental effects on the cell. Downregulation, however, may be a more feasible alternative, especially when applied in an inducible fashion, as the expression of genes related to those essential metabolic steps would be only reduced, and not completely abolished. Regulatory tools based on artificial antisense RNA (asRNA) molecules were shown to be useful in heterotrophic microorganisms [11, 12], and some RNA-based tools have already been tested and validated in *Synechocystis* sp. PCC 6803 (hereafter *Synechocystis*) [13–15]. One of those regulatory tools is the Hfq-MicC system from *Escherichia coli* [16], which was adapted for *Synechocystis* sp. PCC 6803 by Sun et al*.* [17]. This system relies on the expression of a synthetic asRNA, which is complementary to the translational start site of the target gene, and the Hfq chaperone protein. Once bound to the asRNA, the chaperone promotes the annealing of the asRNA to the target mRNA and recruits endoribonucleases for degradation of the target, while conferring protection of the asRNA from cleavage.

**Fig. 1:**
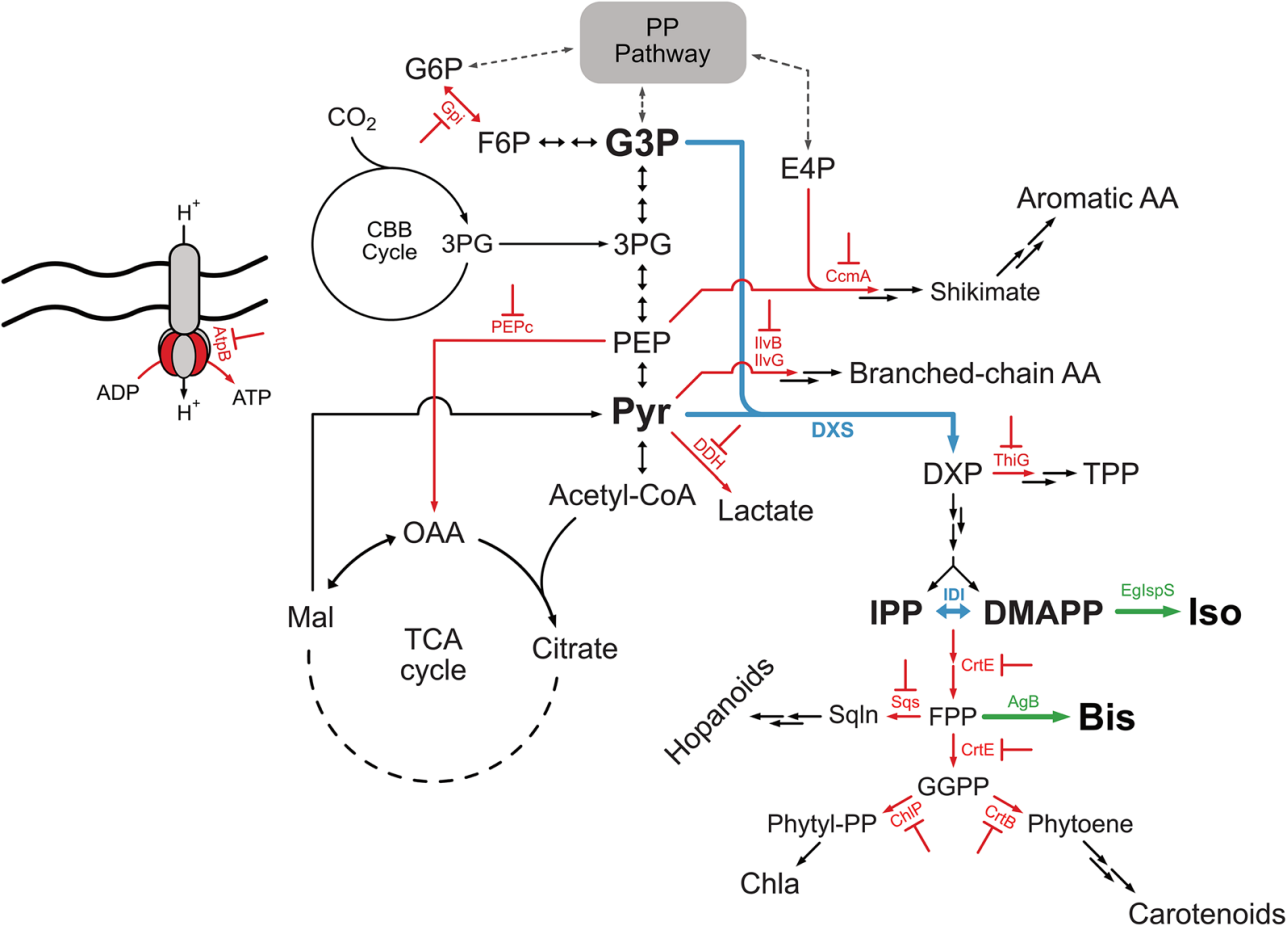
Schematic representation of the metabolic pathway of a cyanobacterial cell, engineered to produce either isoprene or bisabolene, and the steps chosen as targets for gene knockdown (in red). Blue arrows represent enzymatic steps which are present in the native metabolism but were overexpressed for enhanced terpenoid production. Green arrows represent heterologous expression of isoprene synthase from *Eucalyptus globulus* and bisabolene synthase from *Abies grandis* for isoprene and bisabolene production, respectively. PP: pentose phosphate; CBB: Calvin Benson Bassham; TCA: tricarboxylic acid; G6P: glucose 6-phosphate; F6P: fructose 6-phosphate; G3P: glyceraldehyde 3-phosphate; E4P: erythrose 4-phosphate; 3PG: 3-phosphoglycerate; PEP: phosphoenolpyruvate; AA: amino acids; Pyr: pyruvate; Acetyl-CoA: acetyl coenzyme A; OAA: oxaloacetate; Mal: malate; DXP: deoxy-d-xylulose 5-phosphate; TPP: thiamine pyrophosphate; IPP: isopentenyl pyrophosphate; DMAPP: dimethylallyl pyrophosphate; FPP: farnesyl pyrophosphate; GGPP: geranylgeranyl pyrophosphate; Sqln: squalene; Chl*a*: chlorophyll *a*; Iso: isoprene; Bis: bisabolene; Phytyl-PP: phytyl pyrophosphate; ADP: adenosine diphosphate; ATP: adenosine triphosphate; DXS: Deoxy-d-xylulose-5-phosphate synthase; IDI: IPP:DMAPP isomerase; Gpi: glucose 6-phosphate isomerase; CcmA: 3-Deoxy-d-arabinoheptulosonate 7-phosphate (DAHP) synthase; PEPc: PEP carboxylase; IlvB /IlvG: catalytic subunit isoforms of acetolactate synthase; DDH: d-lactate dehydrogenase; ThiG: thiazole synthase; CrtE: polyprenyl transferase; Sqs: squalene synthase; EgIspS: isoprene synthase from *Eucalyptus globulus*; AgB: bisabolene synthase from *Abies grandis*; ChlP: geranylgeranyl pyrophosphate reductase; CrtB: phytoene synthase; AtpB: β-subunit of ATP synthase

In this study, we sought to use the Hfq-MicC tool to knockdown genes in *Synechocystis* that encode key enzymes involved either in the biosynthesis of the native terpenoids or in the central carbon metabolism to try to identify the major competing pathways for heterologous terpenoid production (see Fig. 1 for a general view of the metabolic steps targeted). The set of target genes chosen for knockdown comprises genes encoding enzymes from native terpenoids biosynthesis (*crtE, chlP, sqs, crtB*), thiamine production (*thiG*), central carbon metabolism (*gpi, pepc, ddh*), amino acid biosynthesis (*ccmA, ilvB and ilvG*) and ATP biosynthesis (*atpB*) (see Fig. 1 and Table 1). Isoprene was chosen as the main reporter molecule for this study, not only because it is directly derived from one of the MEP pathway products (DMAPP), but also because its volatile nature allows it to easily escape to the extracellular environment [18]. Bisabolene, a 3-prenyl-unit terpenoid, was also used to evaluate if the patterns observed in isoprene production when interfering with parts of the cyanobacterial terpenoid metabolism hold for the production of longer terpenoids.

**Table 1:** List of genes chosen in this study as targets for knockdown

Gene name	Gene	Encoded protein	Substrate	Pathway
*crtE*	*slr0739*	Polyprenyl transferase	IPP + DMAPP	Terpenoids
*chlP*	*sll1091*	Chlorophyll synthase	GGPP	Chlorophyll *a*
*crtB*	*slr1255*	Phytoene synthase	GGPP	Carotenoids
*sqs*	*sll0513*	Squalene synthase	FPP	Hopanoids
*thiG*	*slr0633*	Thiamine synthase	DXP	Vitamin B_1_
*ddH*	*slr1556*	d*-*Lactate dehydrogenase	Pyruvate	Lactate
*pepc*	*sll0920*	Phosphoenolpyruvate carboxylase	PEP	Central-carbon metabolism
*atpB*	*sll1324*	β-subunit of ATP synthase	ADP + Pi	ATP synthesis
*gpi*	*slr1349*	Glucose 6-phosphate isomerase	F6P/G6P	Sugar metabolism
*ilvB* *ilvG*	*sll1981* *slr2088*	Acetolactate synthase large subunit Acetolactate synthase large subunit	Pyruvate	Branch-chain amino acids
*ccmA*	*sll0934*	Deoxy-d-arabinoheptulosonate 7-phosphate (DAHP) synthase	E4P and PEP	Aromatic amino acids

## Results

### Characterization of an isoprene-producing base strain

In order to identify possible biosynthetic pathways that impose significant constraints on terpenoid production in *Synechocystis*, we sought to target genes that encode enzymes involved in several branches of the metabolism, using an antisense RNA regulatory tool for gene knockdown and isoprene as reporter molecule. Using a heterologously expressed reporter rather than observing effects on native cyanobacterial pigments helps insulating the effectiveness of the asRNAs from native regulation. Isoprene is a small terpene formed from DMAPP in a reaction catalysed by the isoprene synthase enzyme. This terpene was chosen to be used as reporter molecule in this study for several reasons: (i) isoprene is synthesized directly from one of the products of the MEP pathway; (ii) its volatile nature allows it to easily escape the cells in a gaseous form; (iii) isoprene itself is a terpenoid with high industrial relevance; (iv) its biosynthesis has already been introduced and characterized in *Synechocystis* [18–20].

Only a single enzyme, an isoprene synthase, is required to be expressed to achieve isoprene biosynthesis in *Synechocystis*. However, and according to previous studies on the characterization of the MEP pathway conducted by Englund et al. [18] the heterologous expression of more efficient isoprene synthases is challenging, likely because it shifts the IPP:DMAPP ratio in the cells due to its consumption of DMAPP, and thus leads to a metabolic burden on the cells [18]. The authors demonstrated that overexpressing the first (Deoxy-d-xylulose 5-phosphate synthase, DXS) and last (IPP:DMAPP isomerase, IDI) enzymes of the MEP pathway (Fig. 1) overcomes the burden imposed by the isoprene synthase and render *Synechocystis* cells with higher isoprene productivities. The effect of overexpressing these two enzymes from the MEP pathway was also shown fruitful in another study on isoprene production in *Synechococcus elongatus* PCC 7942, performed by Gao et al*.* [21].

**Fig. 2:**
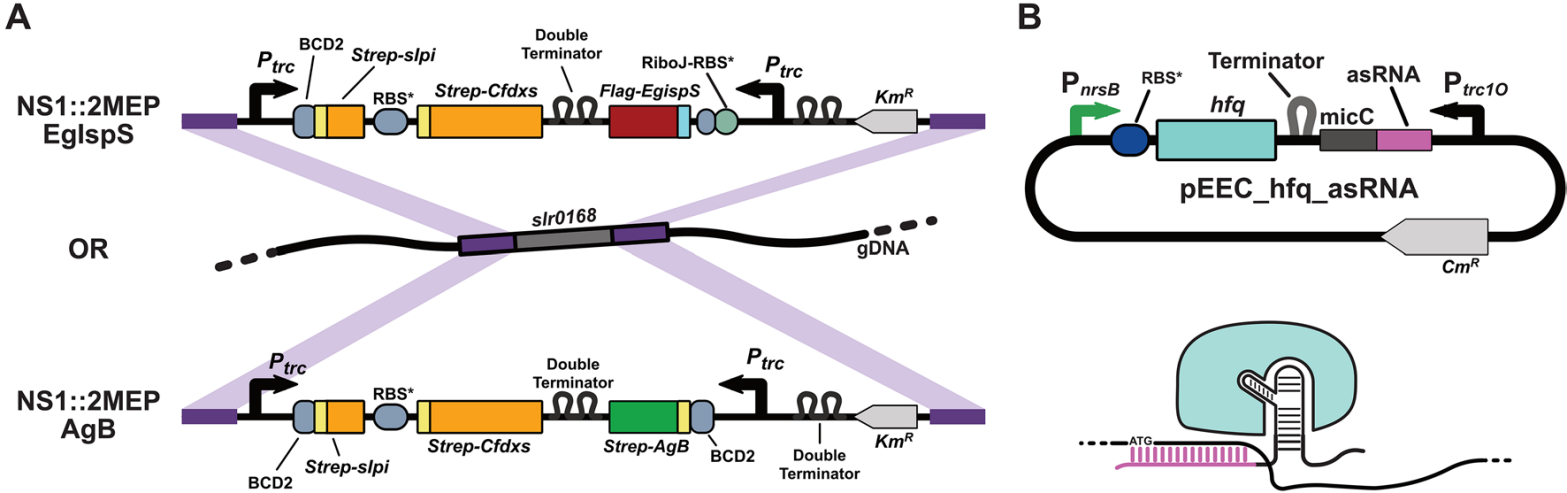
Schematic representation of the synthetic devices integrated in the cyanobacterial chromosome (**A**) for production of either isoprene (top [22]) or bisabolene (bottom), as well as the self-replicating vector containing the Hfq-MicC asRNA regulatory tool and the Hfq-asRNA complex bound to the target mRNA (**B**)

We therefore started by characterizing an isoprene-producing *Synechocystis* strain, generated in another study [22], which presents stable expression and consistent productivity, to be used as a base strain for the gene knockdown experiments. This strain over-expresses DXS from *Plectranthus barbatus*, native *Synechocystis* IDI and the isoprene synthase from *Eucalyptus globulus*, for higher production of isoprene [18, 21, 23, 24], with the genes integrated in the *slr0168* locus, described as a neutral site in the cyanobacterial chromosome [25] (Fig. 2A).

**Fig. 3:**
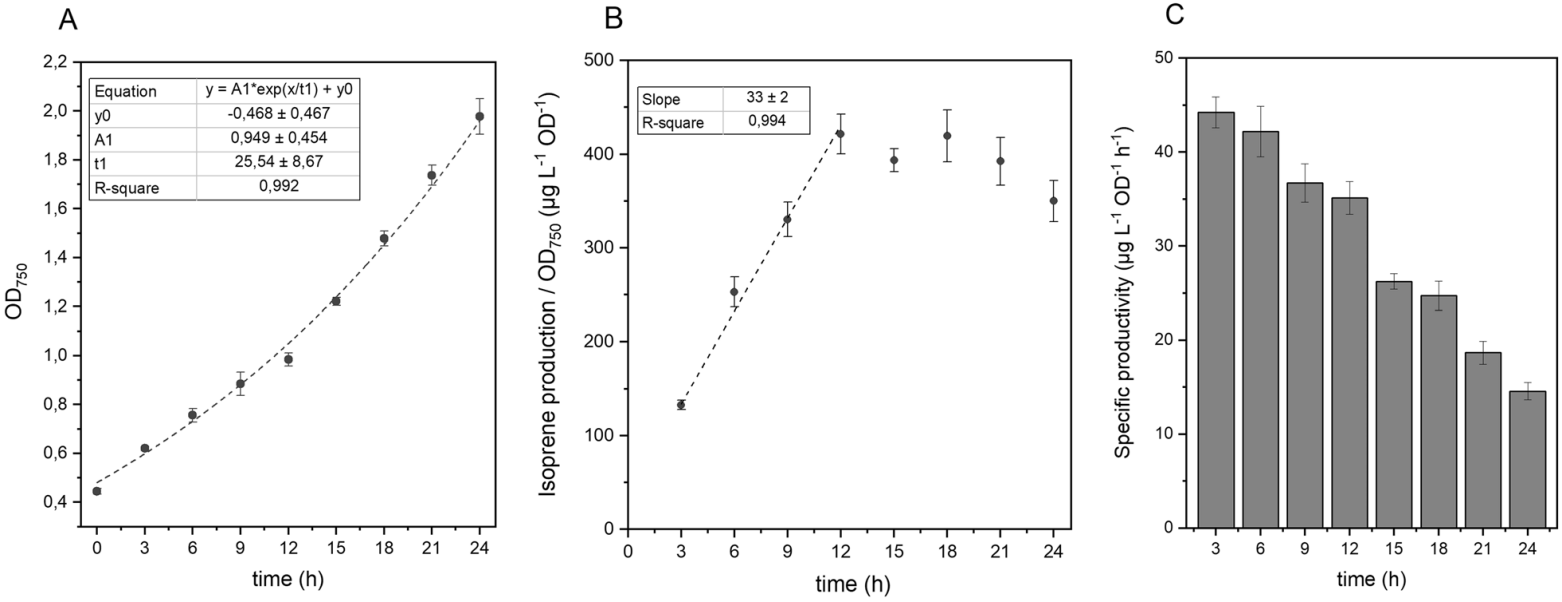
Growth (**A**), isoprene production per cell (**B**) and specific isoprene productivity (**C**) of the NS1::2MEP-EgIspS *Synechocystis* strain over the course of 24 h of cultivation in closed system

Cultures of the resulting strain, termed NS1::2MEP-EgIspS, were grown in media buffered to pH = 8.0 and supplemented with 50 mM NaHCO_3_ in Erlenmeyer flasks for 3 days, and then the performance of the strain for isoprene production was assessed in closed vials over the course of one day. The growth of the cultures and the isoprene content in the headspace of the tubes were evaluated at different discrete time points. The NS1::2MEP-EgIspS base strain presented an exponential growth pattern when cultivated for 24 h in closed vials (Fig. 3A) and a linear increase in the isoprene produced per cell over the first 12 h of cultivation (Fig. 3B). Incubating this strain in closed vials for 3 or 6 h did not influence its specific productivity, and incubation periods of either 9 or 12 h resulted in a minor decrease (roughly 10% compared to 3- or 6-h incubations) in the performance of the strain (Fig. 3C). Interestingly, this linear behaviour in isoprene production was no longer observable for incubation periods longer than 12 h, with more pronounced decreases in the specific productivity (Fig. 3B and C). Furthermore, an increase in the pressure inside the culture tubes was observed beyond 12 h of cultivation, resultant from a build-up of molecular oxygen from the photosynthetic activity and, to a minor extent, to the accumulation of isoprene produced by the cells.

### Testing efficiency of Hfq-asRNA system by targeting *crtE*

In the study conducted by Sun et al*.*, an antisense RNA genetic tool was adapted for gene knockdown in *Synechocystis* [17]. This tool relies on the expression of the Hfq chaperone from *Escherichia coli* and an antisense RNA (asRNA) fused to the *micC* RNA scaffold. When combined, the Hfq-asRNA complex interferes with mRNA translation and promotes target RNA degradation [16]. For controlled activation of the synthetic tool, we choose in our constructs to express the *hfq* gene under regulation of the *nrsB* nickel-inducible promoter. In order to test this modified version of the synthetic tool, as well as to find the best conditions for the subsequent studies, the enzyme encoded by the *crtE* gene (*slr0739*) was chosen as a target for downregulation. CrtE is the sole enzyme in *Synechocystis* responsible for the biosynthesis of polyprenyl backbones [26, 27]. This enzyme catalyses the sequential elongation of terpene backbones by condensation of IPP units, first to DMAPP and then to a polyprenyl pyrophosphate molecule [27], to generate geranyl pyrophosphate (GPP), farnesyl pyrophosphate (FPP) and geranylgeranyl pyrophosphate (GGPP) [7, 27]. It has also been shown that knockdown of *crtE* via CRISPRi led to a decrease in the native carotenoid pool [28].

**Fig. 4:**
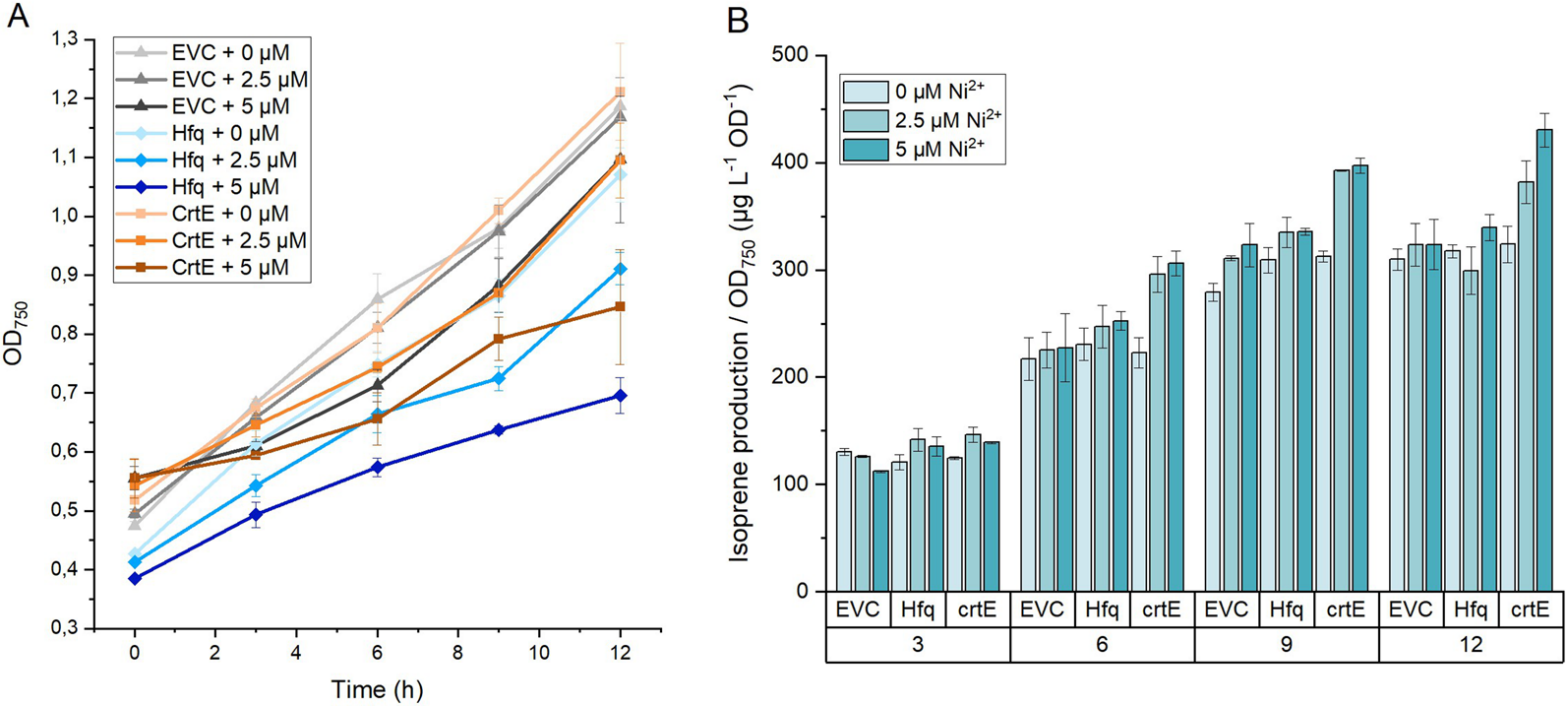
Growth (**A**) and isoprene production normalized to the OD at 750 nm (**B**) of the ISO_EVC, ISO_Hfq and ISO_CrtE, grown in closed vials for 3, 6, 9, and 12 h and in the presence of 0, 2.5 or 5 µM NiCl_2_

The open reading frame encoding Hfq was amplified from *E. coli* and cloned in an RSF1010-based self-replicating vector together with the P_*nrsB*_ promoter. The reverse complementary sequence of the translation start site of *crtE* was combined to the *micC* RNA scaffold sequence and cloned together with the strong P_*trc1O*_ promoter [29] and the *rbcL* terminator [17] into the plasmid carrying the P_*nrsB*_-hfq device (see Fig. 2B). The assembled plasmid was transformed into NS1::2MEP-EgIspS *Synechocystis* cells, resulting in the engineered strain ISO_crtE. This strain was then grown in parallel with two control strains—ISO_EVC, containing an empty self-replicating vector, and ISO_Hfq, containing the P_*nrsB*_-hfq device without any asRNA—in the presence of different concentrations of nickel and over different cultivation periods in closed systems. Pre-cultures of the three strains were exposed for 24 h to 0, 2.5 and 5 µM NiCl_2_ and then cultivated in closed vials in the same concentrations of inducer for 3, 6, 9 and 12 h. The growth of ISO_EVC was only affected when grown at 5 µM of NiCl_2_, presenting the same growth pattern when grown either at 0 or 2.5 µM NiCl_2_ (Fig. 4A). On the other hand, 2.5 µM of NiCl_2_ was enough to cause growth impairment to the ISO_Hfq strain. Regarding isoprene production per cell, all strains produced the same amounts of isoprene when cultivated in the absence of the inducer (Fig. 4B).

An incubation of the ISO_CrtE strain for 3 h in closed vials in the presence of NiCl_2_ was not enough to detect any difference in the isoprene concentration in the headspace compared to the control. However, addition of nickel to the ISO_crtE culture resulted in an increased production of isoprene per cell after 6 h of incubation, and the amounts accumulated in the headspace of the vials were higher than any of the controls, regardless of the nickel concentration used (Fig. 4B). We thus confirmed that interfering with *crtE* expression leads to improvements in isoprene productivity, and that a NiCl_2_ concentration of 2.5 µM is enough for appropriate activation of the Hfq-MicC tool. Furthermore, adding 5 µM of NiCl_2_ did not contribute to any improvements in terms of isoprene production per cell at earlier time points, but led to impaired growth in all strains. Therefore, we decided to conduct our exploratory study using a NiCl_2_ concentration of 2.5 µM for inactivation of all other target genes. Regarding incubation time in closed vials, we chose an incubation period of 6 h for the next tests, as it was enough to lead to observable changes in the isoprene titres. An incubation period of 9 h would also be appropriate for these tests; however, considering the more pronounced effect of 2.5 µM nickel on the growth of ISO_Hfq control, we decided to use the shortest incubation period possible to have less variability between controls in terms of growth. Additionally, measuring at 6 h allows a more selective screening, as only the most effective targets would be selected at this time point.

### Targets chosen for knockdown regulation

Once we had tested the Hfq-MicC tool with the *crtE* gene and established the best conditions to assess the effect of downregulation of different branches of the metabolic network on the terpenoid biosynthetic pathway, we chose a set of genes encoding key enzymes from several different pathways that are related to the pyruvate and G3P pools in *Synechocystis* (Table 1 and Fig. 1) to also downregulate using the same technique. We chose twelve genes encoding enzymes related to the MEP pathway and terpenoid biosynthesis, to the central carbon metabolism and pyruvate consumption, to amino acid formation, or to general bioenergetics, thereby covering several relevant branches of the native metabolism.

### MEP pathway & terpenoid biosynthesis

When envisioning *Synechocystis* as a platform for the heterologous production of terpenoids, improving the availability of the prenyl units specifically towards the compound of interest via downregulation of the native terpenoid biosynthesis may be the first option that comes to mind. The majority of the IPP and DMAPP generated is used for the biosynthesis of carotenoids and the phytol tail of chlorophyll *a* [7]. Carotenoids are all synthesised from phytoene, which in turn is obtained via the condensation of two GGPP molecules catalysed by the phytoene synthase, CrtB [30]. GGPP is also reduced to phytyl pyrophosphate by the GGPP reductase ChlP, and used in synthesis of chlorophyll *a* [31]. We therefore decided to target the genes that encode the GGPP reductase (*chlP*, *sll1091*) and phytoene synthase (*crtB*, *slr1255*). Besides chlorophyll *a* and carotenoids, *Synechocystis* also synthesizes hopanoids, compounds analogous to the plant sterols that play an important role in the fluidity of the biological membranes. Hopanoids are obtained from the 30-carbon-atom terpene squalene, which in turn is synthesized via the condensation of two FPP molecules catalysed by the enzyme squalene synthase [32, 33]. The gene that encodes the squalene synthase (*sqs*, *sll0513*) was also chosen as target for knockdown [34].

The first step of the MEP pathway, catalysed by DXS, corresponds to the irreversible decarboxylation of pyruvate and its condensation with G3P to form deoxy-d-xylulose 5-phosphate (DXP) [7]. DXP is also used as a substrate for synthesis of thiamine pyrophosphate (TPP), a cofactor involved in many catalytic reactions including the one catalysed by DXS [7, 35]. The gene encoding the thiamine synthase (*thiG*, *slr0633*) was thus included as target for knockdown.

### Central-carbon metabolism

Pyruvate is one of the central carbon compounds used as substrate for the biosynthesis of many different cellular metabolites. Lactate is one of these products: it can be generated directly from the reduction of pyruvate by the enzyme lactate dehydrogenase [36]. Given the direct relationship of this dehydrogenase to the pyruvate consumption, we also included the gene encoding lactate dehydrogenase (*ddh*, *slr1556*) in this exploratory study.

A previously reported flux balance analysis (FBA) indicated a possible increase in isoprene production when reducing the carbon flow through the step catalysed by phosphoenolpyruvate carboxylase (PEPc) [18]. PEPc catalyses the carboxylation of phosphoenolpyruvate into oxaloacetate, connecting glycolysis with the TCA cycle. In light of those results, we also targeted the gene that encodes PEPc (*pepc*, *sll0920*) for knockdown.

Both pyruvate and G3P are intermediates in glycolysis and gluconeogenesis. *Synechocystis* cells accumulate carbohydrates such as glycogen as carbon storage when grown under photoautotrophic conditions [37]. In order to evaluate whether decreasing sugar anabolism can improve carbon reallocation towards terpenoid biosynthesis, we decided to downregulate the last step of gluconeogenesis, the isomerization of fructose 6-phosphate (F6P) into glucose 6-phosphate (G6P), as targeting any other step closer to the MEP pathway could also affect the pentose phosphate pathway. Therefore, the gene encoding glucose 6-phosphate isomerase (*gpi*, *slr1349*) was also included in the list of target genes.

### Amino acid biosynthesis

Besides carbohydrates, amino acids also constitute a substantial fraction of biomass and some of them derive directly from pyruvate, such as the branched-chain amino acids. The enzyme acetolactate synthase (AlsS) uses TPP to combine two molecules of pyruvate into one molecule of acetolactate, which then serves as substrate for the biosynthesis of valine, leucine and isoleucine [38, 39]. This enzyme is encoded by the genes *ilvN*, *ilvB* and *ilvG* in *Synechocystis* [40]. The genes *ilvB* (*sll1981*) and *ilvG* (*slr2088*), are annotated as encoding the catalytic subunit of AlsS and were therefore chosen as targets. Aromatic amino acids are synthesised from the shikimate pathway, which utilizes phosphoenolpyruvate and erythrose 4-phosphate, key metabolites from the glycolytic pathway [41]. In *Synechocystis*, the gene that encodes the first enzyme of the shikimate pathway was incorrectly annotated as a carboxysome-forming protein (*ccmA*, *sll0934*) [42], but studies have demonstrated that this protein is instead involved in aromatic amino acid production [43] and therefore we chose to include it in this study.

### Bioenergetics

As pointed out by previous studies, the ratio between ATP and NAD(P)H concentrations is an important factor to take in consideration when engineering cyanobacteria for microbial production, including for terpenoids, where an increase in the NADPH:ATP ratio would be beneficial for the terpenoid biosynthetic pathway [18, 44]. Therefore, we included the gene encoding the β-subunit of ATP synthase (*atpB*, *sll1324*) in our set of genes for knockdown.

### Assessing the effect of gene knockdown on isoprene production

**Fig. 5:**
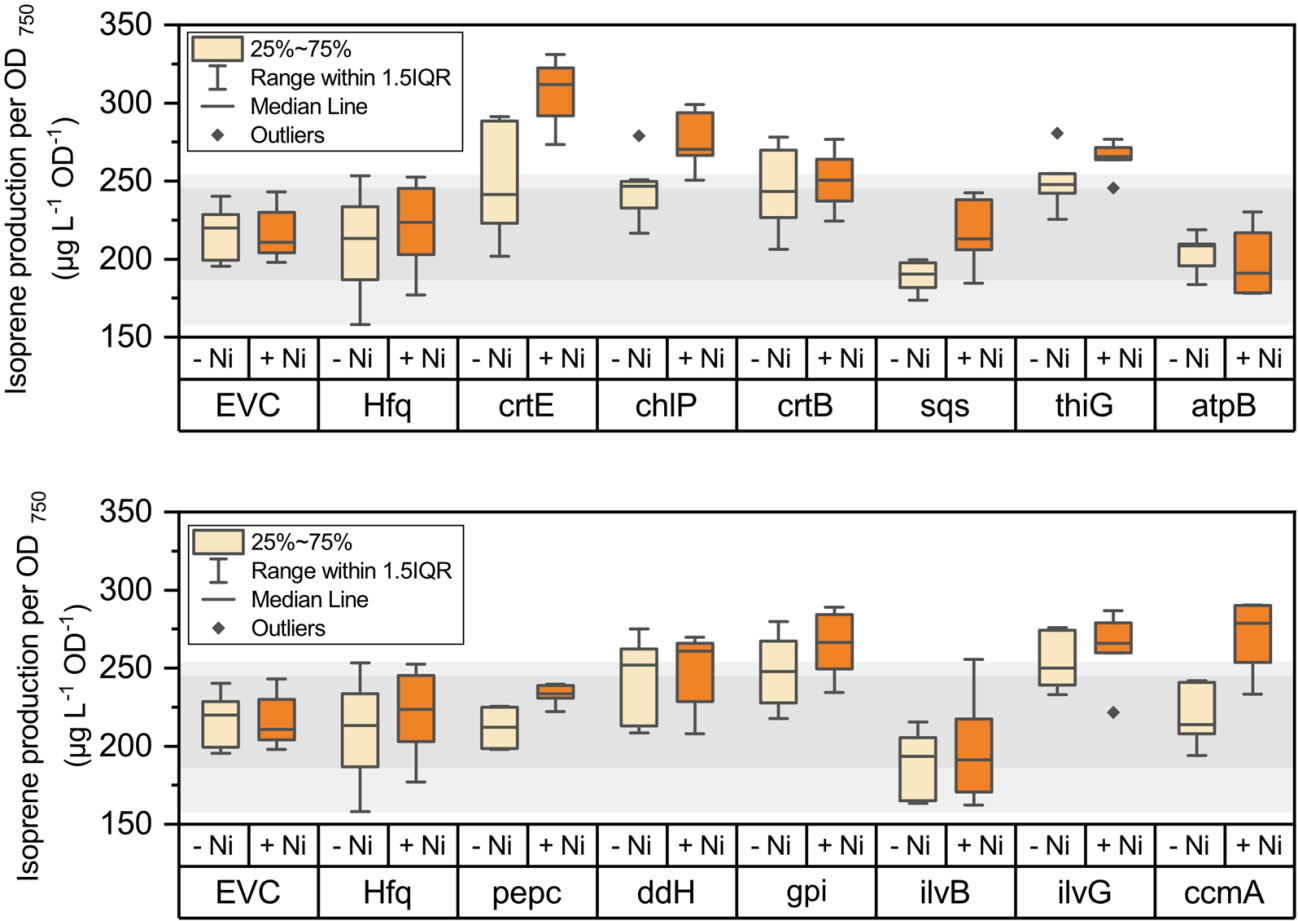
Isoprene production per cell of all strains generated after 6 h of incubation in closed vials, in the absence of nickel or in the presence of 2.5 µM NiCl_2_. The results were split into two panels for better visualization. In each panel, the isoprene production of both control strains was also included. Light grey area represents the production range observed for all samples of both controls, while the dark grey area represents the 25–75% range of the controls. All boxes represent values of at least six replicates from at least two independent experiments

After selecting genes as targets for downregulation, we generated Hfq-MicC systems for individual gene knockdown in the same way as performed for *crtE.* For all cases, the asRNA-micC sequence was assembled with the strong constitutive P_*trc1O*_ promoter, cloned into the self-replicating plasmid containing the *hfq* gene under control of the P_*nrsB*_ promoter and the resulting plasmids were transformed into the NS1::2MEP-EgIspS *Synechocystis* strain. The performance of each isoprene-producing strain was then tested using the method optimized with ISO_crtE, as described in section "Testing efficiency of Hfq-asRNA system by targeting *crtE*". Isoprene and growth were assessed in cultures grown for 6 h in closed vials either without induction or induced with 2.5 µM NiCl_2_. The isoprene production per cell of each strain, with or without nickel induction, is shown in Fig. 5.

Comparing the performance of all engineered strains and to the performance of the controls, six candidates presented a clear positive effect on isoprene production per cell. From these six candidates, three of them, *crtE*, *chlP* and *thiG*, are related to the MEP pathway and biosynthesis of native terpenoids, while two, *ilvG* and *ccmA*, are related to amino acid production and one, *gpi*, is related to sugar catabolism (Fig. 5). In the case of knockdown of *ilvG*, we note that there is no statistically significant variation between induced and uninduced states of this strain (Fig. 5, lower panel), suggesting that either this asRNA has already some effect on the cells even without the presence of Hfq, or leakiness of the P_*nrsB*_ promoter is enough to drive the knockdown of *ilvG* expression. Nonetheless, the uninduced ISO_ilvG is not statistically different from the ISO_Hfq control, which indicates that the gene knockdown of *ilvG* via induction of Hfq-MicC system contributed to the observed positive effect on the isoprene titres. A similar pattern was observed for ISO_gpi (Fig. 5, lower panel), where there was no significant difference between production of the culture in the absence or presence of NiCl_2_, but only the induced cultures presented a statistically significant difference from the controls. Concerning bioenergetics, we did not observe any variation upon induction of the ISO_atpB strain, and isoprene production was comparable to the controls (Fig. 5, upper panel).

**Fig. 6:**
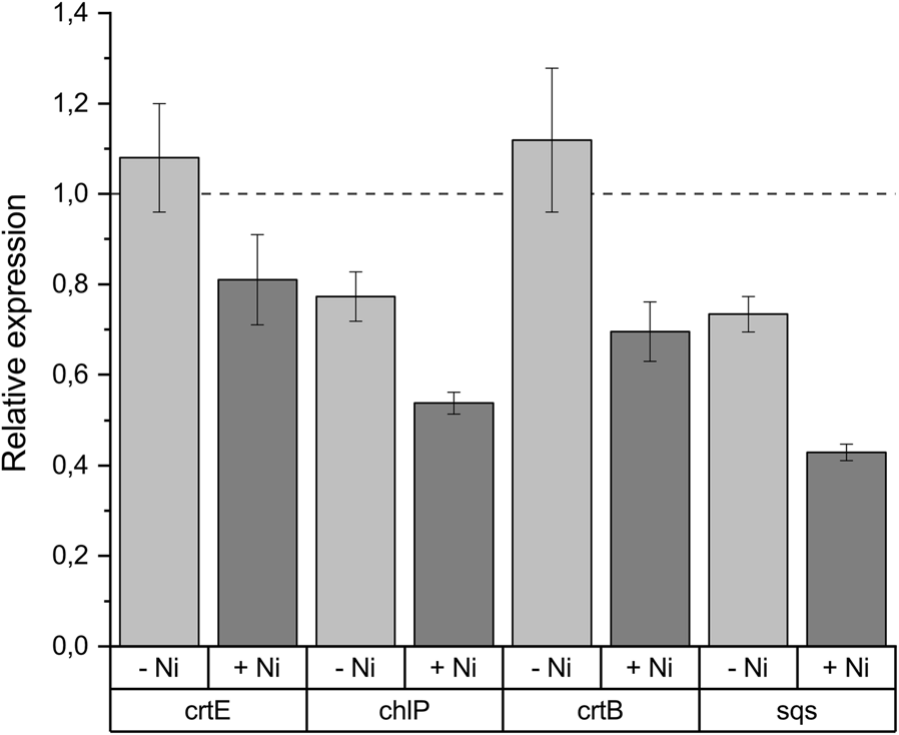
RT-qPCR for the relative expression of *crtE, chlP, crtB* and *sqs* genes in ISO_crtE, ISO_chlP, ISO_crtB and ISO_sqs strains, respectively, compared to the levels of ISO_EVC. Error bars represent standard deviation of three technical replicates for each sample

To confirm that our Hfq-MicC systems are working in the isoprene-producing engineered strains, we evaluated the transcript levels of the target genes in two strains that presented a positive correlation between gene knockdown and isoprene production—ISO_crtE and ISO_chlP—as well as two strains that did not present differences when compared to the controls—ISO_crtB and ISO_sqs. We observed that *chlP* and *sqs* transcription was already repressed by 20 and 30% in the absence of nickel, respectively, when compared to ISO_EVC; but upon induction both genes were knocked down by *ca.* 50% (Fig. 6). Similarly, induction in ISO_crtE and ISO_crtB led to 20% and 30% decrease in the transcript levels of *crtE* and *crtB*, respectively, demonstrating that the Hfq-MicC tool is functional in our strains, also for some genes where knockdown did not result in improved isoprene production performance.

### Bisabolene production & interference of putative competing pathways

**Fig. 7:**
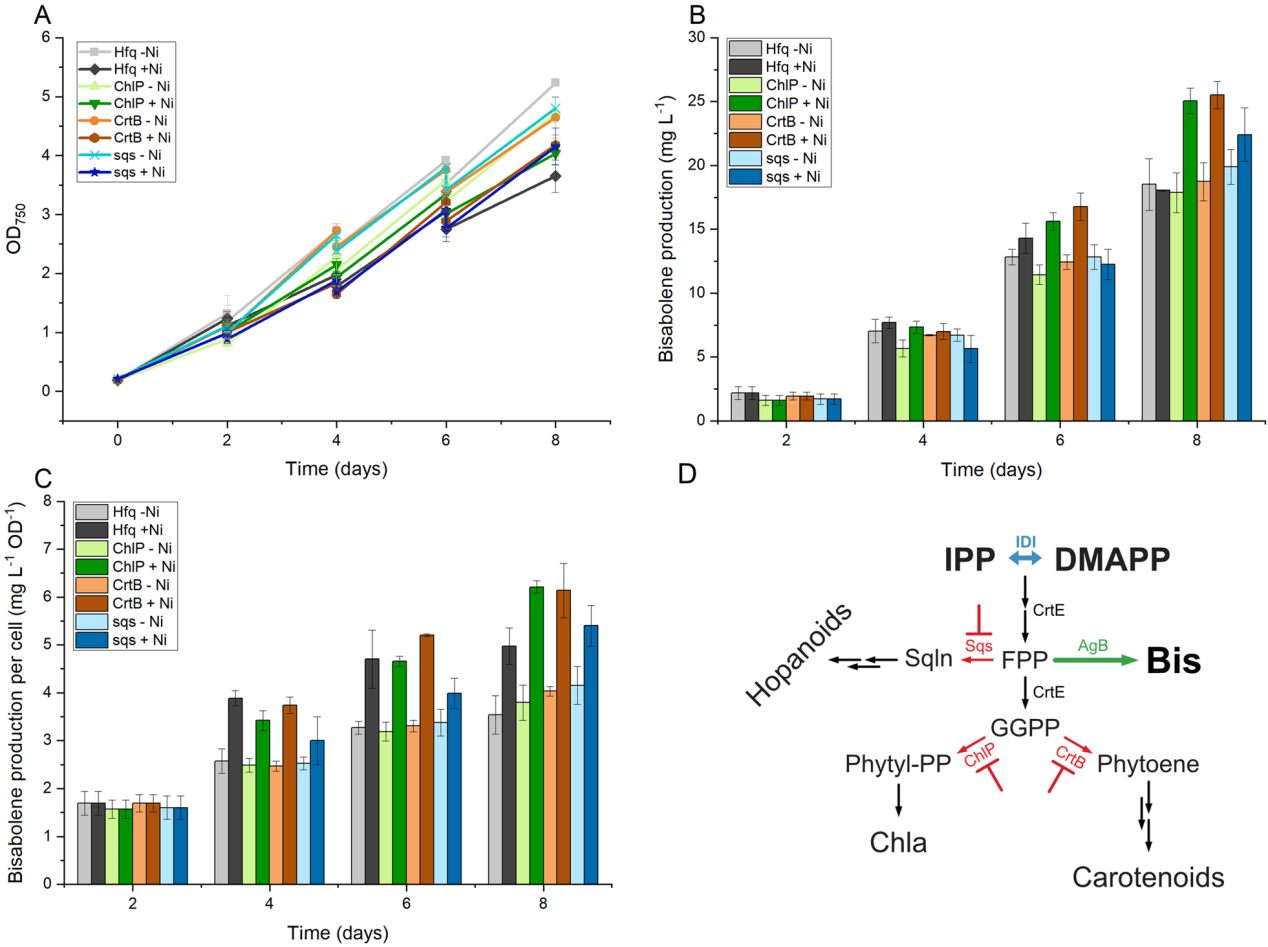
Growth (**A**), bisabolene cumulative production (**B**) and specific bisabolene production (**C**) of BIS_Hfq, BIS_chlP, BIS_crtB and BIS_sqs over the course of 8 days of cultivation with 0 and 3 µM of NiCl_2_, as well as schematic representation of the terpenoid biosynthetic pathway of the bisabolene-producing cyanobacteria and target genes for downregulation (**D**). IPP: isopentenyl pyrophosphate; DMAPP: dimethylallyl pyrophosphate; FPP: farnesyl pyrophosphate; GGPP: geranylgeranyl pyrophosphate; Sqln: squalene; Bis: bisabolene; Phytyl-PP: phytyl pyrophosphate; Chl*a*: chlorophyll *a*; IDI: IPP:DMAPP isomerase; CrtE: polyprenyl transferase; Sqs: squalene synthase; AgB: bisabolene synthase from *Abies grandis*; ChlP: geranylgeranyl pyrophosphate reductase; CrtB: phytoene synthase

After evaluating the effect of gene knockdown on isoprene production, we decided to test the same approach but for a different terpenoid with a longer carbon chain—bisabolene. In contrast to isoprene, which is produced directly from one molecule of DMAPP, bisabolene is a sesquiterpenoid and, thus, its biosynthesis from FPP requires two molecules of IPP and one of DMAPP. Considering that FPP is also a substrate for the biosynthesis of squalene, as well as for GGPP, the precursor of all carotenoids and the phytol tail of quinones, chlorophyll *a* and tocopherols, we decided to assess whether knocking down genes related to the native terpenoid biosynthesis would be more effective than for shorter molecules such as isoprene. We therefore generated a bisabolene-producing base strain with a synthetic device containing a codon-optimized version of the gene encoding bisabolene synthase from *Abies grandis* under control of the strong constitutive P_*trc*_ promoter. The *dxs-idi* operon used in the isoprene base strain was also included. This synthetic device was integrated in the *slr0168* locus, and the resulting engineered strain was named NS1::2MEP-AgB (Fig. 2A). The Hfq-MicC systems targeting either *chlP*, *crtB* or *sqs* were transformed into this base strain, and a control was generated by transforming NS1::2MEP-AgB with the pEEC_P_*nrsB*_-hfq plasmid (see Tables 1 and 2 and Fig. 7D for details on the strains). Cultures of these four strains were cultivated in Erlenmeyer flasks for 8 days at 30 °C with constant illumination. An organic layer of dodecane was added to each culture, to trap the produced bisabolene [8]. On the second day of cultivation, NiCl_2_ was added at a final concentration of 3 µM to induce the Hfq-MicC systems. Control cultures were grown without induction. Figure 7 summarizes the results obtained for bisabolene production.

**Table 2:** List of plasmids used in this study

Plasmid	Relevant characteristics	Type of plasmid	References
pEEC	Self-replicating plasmid, derived from pPMQAK plasmid (RSF1010 based); Chloramphenicol resistance	Self-replicating	[18]
pEERM3	Integrative plasmid, targets *slr0168* neutral site of *Synechocystis* genome; Kanamycin resistance	Integrative	[23]
p6EgIspS	Self-replicating plasmid, carrying the codon-optimized versions of *dxs* and *idi* genes from *Coleus forskohlii* and *Synechocystis* sp. PCC 6803, respectively, in an operon controlled by the constitutive P*trc* promoter and a bicistronic device; chloramphenicol resistance	Self-replicating, derived from pEEC	[18]
pAgBispA	plasmid carrying codon-optimized gene encoding *Abies grandis* bisabolene synthase, under control of the P_*trc*_ promoter	Self-replicating, derived from pEEC	[2]
pEEC_P_*nrsB*_-hfq	plasmid carrying hfq gene from *E. coli* under control of the Nickel-inducible P_*nrsB*_ promoter and RBS*	Self-replicating, derived from pEEC	This study
pEERM3_2MEP	Integrative plasmid (targets *slr0168* neutral site of *Synechocystis* genome) carrying the codon-optimized versions of *dxs* and *idi* genes from *Coleus forskohlii* and *Synechocystis* sp. PCC 6803, respectively, in an operon controlled by the constitutive P*trc* promoter and a bicistronic device. Kanamycin resistance	Integrative, derived from pEERM3	This study
pEERM3_2MEP-AgB	Integrative plasmid (targets *slr0168* neutral site of *Synechocystis* genome) the 2 MEP genes as an operon, as well as the bisabolene synthase gene under control of the constitutive Ptrc promoter and a bicistronic device. Kanamycin resistance	Integrative, derived from pEERM3_2MEP	This study
pEEC_Hfq_ X	plasmid carrying both the hfq cassette, controlled by PnrsB promoter, and the antisense-RNA cassette, controlled by the constitutive P_*trc1O*_ promoter; X can be *ccmA, crtB, chlP, citS, pdhB, pepC, ddH, gpi, sqs, thiG, ilvB, ilvG* or *atpB*	Self-replicating, derived from pHfq	This study

Regarding growth, all strains presented the same behaviour in the absence of nickel (Fig. 7A). Addition of NiCl_2_ led to growth impairment in all strains; however, the BIS_Hfq strain presented a more pronounced reduction in growth, demonstrating that expression of the chaperone alone has a negative impact on the cell physiology. This impact of Hfq on the engineered strains is also noticeable in the cell-specific bisabolene production, as an increase in the specific bisabolene titres of the BIS_Hfq strain was observed in presence of the inducer, compared to the uninduced strain (Fig. 7C). Nonetheless, when looking at the cumulative bisabolene production, the control strain presented similar titres regardless of the concentration of NiCl_2_ (Fig. 7B). BIS_sqs did not present any significant variation on bisabolene production compared to the BIS_Hfq control strain when knockdown of the *sqs* gene was induced (Fig. 7B and C). BIS_chlP and BIS_crtB, however, demonstrated an increased performance in bisabolene production, both in terms of volumetric and specific titres (Fig. 7B and C). After 8 days of cultivation, we observed a 20% increase in the bisabolene production of the induced BIS_chlP and BIS_crtB cultures (Fig. 7B).

## Discussion

Here, we have reported the use of an asRNA synthetic tool to target key enzymes of different biosynthetic pathways in *Synechocystis* in order to reveal putative candidates for metabolic engineering for improved terpenoid production. Isoprene, a non-native volatile terpenoid, was used as reporter molecule, as it can be easily measured from the headspace of cultures grown in closed systems and has been already used as reporter molecule in previous studies [18].

The isoprene producing base strain presented a reliable and reproducible behaviour when cultivated over 24 h in closed vials. An observed decrease in the strain productivity after 12 h of cultivation (Fig. 3B) may be related to the increase in the molecular oxygen in the headspace of the culture vials, which may contribute to changes in the photorespiration of the cells and/or lead to increased cellular damage associated to oxidative stress. The increase in culture density over the course of the experiment may also influence productivity, due to a decrease in the light penetration and thus in a lower light availability for photosynthesis.

The Hfq-MicC synthetic tool reported by Sun et al. was chosen as means for knockdown of target genes belonging to different branches of the cyanobacterial metabolism [17], and the nickel-inducible P_*nrsB*_ promoter, identified in previous studies as a promoter with tight regulation and low leakiness [45], was chosen to drive the expression of the Hfq chaperone gene. We observed however, that this tool had a negative impact on the cell physiology in *Synechocystis*, as the induction of Hfq expression in the absence of a partner asRNA resulted in a reduction in growth of the ISO_Hfq strain (Fig. 4A). This effect on the cell physiology, and possibly also the choice of a native promoter subjected to regulatory mechanisms in *Synechocystis*, contributed to an increased variability between replicates and thus a decrease in the sensitivity of the technique. Nonetheless, we were able to observe some trends in the results that correlate gene knockdown with an increase in isoprene productivity, which were consistent over independent experiments (Fig. 5).

From twelve genes chosen as targets for knockdown, we observed a correlation between gene knockdown of *crtE, chlP, thiG, ilvG, ccmA* and *gpi* and enhanced isoprene production, while the other candidate genes presented similar performance as the control strains (Fig. 5). Real-time quantitative PCR of target genes could confirm functionality of the Hfq-MicC system (Fig. 6). A decrease in the relative expression of *crtB* and *sqs*, two targets that did not show any improvements in isoprene production upon induction, was also observed, which demonstrates that Hfq-MicC is not only active in strains that presented an effect on isoprene production. For *chlP* and *sqs*, the gene expression was lower when compared to the ISO_EVC control strain even in the absence of inducer (Fig. 6). This issue was also reported by Sun et al*.* [17]. It is possible that some of the asRNAs used are more stable than others and can affect the gene expression of their target even in the absence of induced levels of Hfq.

Regarding the genes which had an effect on isoprene production upon knockdown, CrtE is the sole enzyme responsible for the elongation of the polyprenyl molecules that serve as precursors for all terpenoids, and our results for the gene are in agreement with the observations by Dietsch et al*.*, where the authors observed that knockdown of *crtE* via CRISPRi led to a decrease in the native carotenoid pool, and its combination with heterologous expression of an FPP synthase led to improvements in the production of the sesquiterpenoid valencene [28]. The *chlP* gene encodes the GGPP reductase, an enzyme that links terpenoid biosynthesis with chlorophyll *a* biosynthesis. Chlorophyll *a* synthesis is one of the major uses of terpenoids in cyanobacteria, and *Synechocystis* is able to recycle chlorophyll molecules by reesterification of the porphyrin with another phytol tail, in a continuous degradation of chlorophyll and reutilization of both porphyrin and phytol molecules [31]. Considering the effect of knocking down *chlP* on isoprene production, it is possible that these recycling pathways suffice to provide the cells with enough chlorophyll *a* for growth, and less IPP and DMAPP is used in phytyl-PP biosynthesis.

ThiG is the enzyme responsible for the first step of thiamine biosynthesis, and shares its substrate DXP with the MEP pathway [35]. Our results here indicate that this pathway has a significant impact on the carbon flux through the MEP pathway and may be a good candidate for further engineering. However, thiamine pyrophosphate is required as cofactor for the DXS catalysis, and thus, one cannot simply eliminate thiamine biosynthesis [7]. A better strategy may be to introduce a bias on DXP utilization towards the terpenoid biosynthesis, possibly by fusing the first and second enzymes of the MEP pathway, DXS and DXR.

The biosynthesis of certain amino acids, such as the branched chain amino acids (BCAA), utilizes pyruvate as substrate. The observed improvements in isoprene production related to gene knockdown of *ilvG*, but not *ilvB*, indicate not only the BCAA pathway as a possible strong competitor with the MEP pathway for pyruvate, but also corroborate the previous suggestions that IlvG is the catalytic subunit of AlsS in *Synechocystis* [39]. The shikimate pathway may also be a strong candidate for downregulation to engineer a higher carbon flux towards terpenoids. DAHP synthase, encoded by *ccmA*, consumes PEP and E4P, which are intermediates from the glycolytic and pentose phosphate pathways, respectively, and constitutes the first step in the shikimate pathway. Although pyruvate is not directly involved in the shikimate pathway, it is generated from PEP via glycolysis. Similarly, E4P formation is tightly connected to G3P, as G3P is a central node in CBB cycle, glycolysis and pentose phosphate pathway (Fig. 1) [39]. Large amounts of carbon are expected to be consumed by this pathway for aromatic amino acid biosynthesis (required for biomass formation), which may influence the carbon flux through the pyruvate and G3P nodes of the metabolic network. Besides protein synthesis, the aromatic amino acids are also involved in the biosynthesis of tocopherols, a class of secondary metabolites found in photosynthetic organisms [46]. Tyrosine is used to generate homogentisate, which is condensed with phytyl-pyrophosphate by the homogentisate phytyltransferase (HPT) to generate the tocopherols [47]. This link between terpenoid biosynthesis and aromatic amino acid biosynthesis may also explain the effect of knocking down *ccmA* on the production of isoprene. Further studies will be required to evaluate whether the effect observed is derived from amino acid formation and/or tocopherol biosynthesis, e.g. by knocking down the gene encoding the HPT. The last target that presented a positive trend towards isoprene production was *gpi*, which encodes the glucose 6-phosphate isomerase enzyme. This enzyme is responsible for the interconversion of fructose 6-phosphate and glucose 6-phosphate and links glycolysis/gluconeogenesis and the pentose phosphate pathways with sugar catabolism [48]. The increased isoprene production per cell upon knocking down *gpi* suggests that the isoprene-producing cell allocates significant amounts of carbon towards sugar storage (e.g. glycogen). However, a study on limonene and bisabolene production in a glycogen-deficient strain of *Synechococcus* sp. PCC 7002 performed by Davies et al*.* did not show any improvements in the terpenoid titres when glycogenesis was interrupted [8]. This suggests that either the cellular metabolism of these two cyanobacteria is different, and eliminating glycogen formation in *Synechocystis* may be more beneficial for terpenoid biosynthesis, or glycogen is not a major competitor to the terpenoid biosynthetic pathway. Alternatively, knocking down *gpi* had a less generally detrimental effect on metabolism than eliminating glycogen formation entirely.

Isoprene is the smallest terpenoid and is directly produced from DMAPP. Producing any other terpene should also benefit from modifications in the targets we identified upstream IPP and DMAPP formation; however, the effect on knocking down reaction steps downstream isoprene biosynthesis may be variable depending on the specific terpenoid or class of terpenoids produced. In order to address this question, we tested gene knockdown of *chlP, crtB* and *sqs* in a bisabolene-producing strain (Fig. 7D). The pattern for *chlP* and *sqs* downregulation remained the same as for isoprene; however, *crtB* knockdown, which did not have any effect on isoprene production, also resulted in an increase in specific bisabolene titres (Fig. 7B and C). In light of these results, we hypothesize that while chlorophyll *a* biosynthesis is a major consumer of GGPP, the balance of GGPP in the cell is affected both by chlorophyll *a* and carotenoids biosynthesis, and decreasing the flux to either of these pathways leads to an increased availability of FPP for bisabolene biosynthesis. Knocking down *sqs* did not affect bisabolene production, possibly because the flux towards hopanoid formation via squalene synthase may be low in the standard laboratory conditions used in our experiment.

## Conclusions

In this study, we have targeted several genes for downregulation using an asRNA synthetic tool and evaluate how decreasing the expression of these genes influences isoprene production in *Synechocystis*, with the aim of mapping pathways of the cyanobacterial metabolic network that may compete with terpenoid biosynthesis for cellular resources. We observed that expression of the chaperone protein Hfq alone imposes stress to *Synechocystis* cells, which decreases the sensitivity of the technique. Nonetheless, we verified the functionality of this tool in *Synechocystis* and we were able to identify six target genes as good candidates for further engineering. Within the native terpenoid biosynthetic pathway, *crtE* was identified as the best candidate for further engineering for enhanced isoprene production. Chlorophyll *a* biosynthesis is also a potentially effective target, while interfering with carotenogenesis only seemed to make a difference for longer terpenes, such as bisabolene, most likely due to shifts between GGPP and FPP availability. Interfering with a competitive pathway that branches out from DXP also led to positive effects on isoprene production, indicating that targeting this branching point may be a good strategy for improved terpenoid production. Finally, other pathways upstream of the MEP pathway, namely amino acid biosynthesis, tocopherol biosynthesis and sugar catabolism, can also be considered good targets for metabolic engineering.

## Materials and methods

### Strains and growth conditions

*Escherichia coli* strain DH5α Z1 (Expressys) was used for subcloning. Cultivation was performed at 37 °C in liquid LB medium supplemented with 35 µg mL^−1^ chloramphenicol or 50 µg mL^−1^ kanamycin, as appropriate.

The isoprene-producing strain NS1::2MEP-EgIspS used here was generated in a previous study [22].

*Synechocystis* cultures were grown from cryo-stocks in BG11 supplemented with either 50 µg mL^−1^ kanamycin (for the isoprene and bisabolene-producing strains) or with 25 µg mL^−1^ kanamycin and 10 µg mL^−1^ chloramphenicol (for the strains transformed with the pEEC_Hfq_asRNA plasmids), in 100 mL Erlenmeyer flasks, at 30 °C and 50 µmol photons m^−2^ s^−1^. These cultures were then used to prepare the seed-cultures for the production experiments.

### Plasmid assembly

The antisense RNA (asRNA) systems were designed and assembled based on the Hfq-MicC genetic tool reported by Sun et al*.* [17], with some modifications. The nickel-inducible P_*nrsB*_ promoter was chosen to drive the expression of the chaperone [45], while the asRNA-micC scaffolds were chosen to be expressed by the constitutive P_*trc1O*_ synthetic promoter [49]. See Fig. 2B for schematic representation of the interference system. The *hfq* coding sequence and the P_*nrsB*_ promoter were amplified by PCR from *E. coli* and the pEERM3 plasmid [23] respectively. The ribosome binding site sequence was added to both the promoter and the *hfq* gene as overhangs, as well as the restriction enzyme binding sites, and the two DNA fragments were combined via Overlap-Extension PCR. The resulting P_*nrsB*_-hfq fragment was cut with EcoRI and PstI and cloned into the pEEC plasmid, previously cut with the same enzymes and dephosphorylated, to form the pEEC_P_*nrsB*_-hfq plasmid.

All 24 bp synthetic asRNA sequences were designed to bind to the translational start site of the chosen genes, with exception of the anti-*crtE* asRNA, which was designed to bind to the ribosome binding site and the first two codons of the *slr0739* (*crtE*) gene. All asRNA sequences were synthesized as gBlock™ gene fragments (Integrated DNA Technologies), together with the P_*trc1O*_ promoter and the *rbcL* terminator. The target genes and the asRNA sequences chosen for this study are summarized in Additional file 1: Table S1. The gBlocks were cut with EcoRI and PstI and cloned into the pEEC_P_*nrsB*_-hfq plasmid, which was previously cut with MunI and PstI and dephosphorylated. The resulting plasmids were confirmed by DNA sequencing prior to their transformation into *Synechocystis* cells.

To generate the bisabolene-producing *Synechocystis* strain, first the operon encoding DXS and IDI was amplified by PCR from the p6EgIspS plasmid and cloned into the pEERM3 plasmid using the EcoRI and XbaI restriction enzymes. The resulting plasmid was designated pEERM3_2MEP (Table 2) Secondly, the bisabolene synthase gene from *Abies grandies* was amplified from the pAgBispA [2] plasmid with a Strep-tag and glycine-serine linker sequence at 5′ as well as restriction enzyme binding sites on both ends. The PCR product was cut with PstI and SpeI and cloned into pEERM3_2MEP, previously cut with PstI and XbaI and dephosphorylated, originating the pEERM3_2MEP-AgB plasmid (Table 2).

All enzymatic digestions were performed with FastDigest enzymes from ThermoScientific, according to their specifications. Dephosphorylation of the plasmid backbones was carried out using FastAP (Thermo Scientific). Ligation reactions were performed using the Quick Ligation kit from New England Biolabs (NEB). PCR amplifications were performed using the Phusion High Fidelity DNA polymerase kit (Thermo Scientific). The full sequence of all the genetic elements used for plasmid assembly in this study are provided in the Additional file 1: Table S1.

### Introduction of gene constructs into *Synechocystis*

The base strain for bisabolene production (NS1::2MEP-AgB) was generated by double homologous-recombination via natural transformation using an optimized protocol previously described [50] (see Table 2 and Fig. 2A). Briefly, *Synechocystis* sp. PCC 6803 cells grown to mid-log phase were collected by centrifugation (10 min at 5000×*g*) and resuspended in fresh medium to a density of 1 × 10^9^ cells mL^−1^. The pEERM3_2MEP-AgB integrative plasmid was added to 400 µL of cell suspension at a final concentration of 10 mg mL^−1^ and the cells were incubated at 50 μmol photons m^−2^ s^−1^ illumination at 30 °C. After 4 to 5 h incubation, the cells were spread on nitrocellulose membranes (GN-6 metricel 47 mm, 0.45 µm, PALL Life Sciences) resting on BG11 agar plates without antibiotics and incubated for another 18 to 24 h under constant 50 μmol photons m^−2^ s^−1^ illumination at 30 °C. For colony selection, the membranes were changed onto new BG11 agar plates with 50 µg mL^−1^ kanamycin and incubated under the same conditions. Single colonies were streaked on a new BG11 agar plate with kanamycin, and were analysed by colony PCR. Positive transformants were inoculated into 6-well cell culture plates (TC plate, Sarstedt) and cultivated in liquid BG11 medium supplemented with kanamycin and propagated continuously until fully segregated. Genomic DNA was extracted using the GeneJET Genomic DNA purification Kit (Thermo Scientific) and the manufacturer specifications for Gram-positive bacteria (as the additional steps lead to improved lysis of the cells), and full segregation was confirmed by PCR using DreamTaq (Thermo Scientific) accordingly to the manufacturer recommendations.

For introduction of the asRNA systems, the plasmid vectors carrying the Hfq-MicC systems were transferred either to NS1::2MEP-AgB or NS1::2MEP-EgIspS *Synechocystis* cells by three-parental mating [29], using the *E. coli* HB101 strain harbouring the pRL443 conjugative plasmid. Single colonies were transferred to BG11 agar plates supplemented with 10 µg mL^−1^ chloramphenicol and 25 µg mL^−1^ kanamycin. The presence of the asRNA systems in *Synechocystis* cells was confirmed by colony PCR. All *Synechocystis* strains used in this study can be found in Table 3.

**Table 3:** List of *Synechocystis* strains used in this study

Strain	Relevant genotypes
NS1::2MEP-AgB	Δ*slr0168*::2MEP-AgB (Km^R^)
NS1::2MEP-EgIspS	Δ*slr0168*::2MEP-EgIspS (Km^R^) [22]
BIS_EVC	Δ*slr0168*::2MEP-AgB (Km^R^) pEEC (Cm^R^)
BIS_Hfq	Δ*slr0168*::2MEP-AgB (Km^R^) pEEC_P_*nrsB*__hfq (Cm^R^)
ISO_EVC	Δ*slr0168*::2MEP-EgIspS (Km^R^) pEEC (Cm^R^)
ISO_Hfq	Δ*slr0168*::2MEP-EgIspS (Km^R^) pEEC_P_*nrsB*__hfq (Cm^R^)
BIS_X*	Δ*slr0168*::2MEP-AgB (Km^R^) pEEC_Hfq_X* (Cm^R^)
ISO_X*	Δ*slr0168*::2MEP-EgIspS (Km^R^) pEEC_Hfq_X* (Cm^R^)

*X corresponds to the name of the target genes (e.g., ISO_crtE corresponds to the isoprene-producing strain with Hfq-MicC system targeting *crtE*)

### Isoprene experiments

Cultures of the isoprene-producing strains (see section "Characterization of an isoprene-producing base strain") were seeded to OD_750_ = 0.1 into 50 mL BG11 supplemented with 50 mM sodium bicarbonate, 50 mM TES buffer (pH adjusted to 8.0) and appropriate antibiotics in 250 mL Erlenmeyer flasks and grown for 3 days under constant white light at 50 µmol photons m^−2^ s^−1^. Then, cells were harvested by centrifugation at 4000×*g* for 10 min, resuspended in fresh medium to OD_750_ = 0.5 and 5 mL of culture were transferred to 8 mL screw cap tubes (Chromacol 10-SV, ThermoFisher Scientific). The cultures were incubated for 3, 6, 9, 12 and 24 h at 30 °C with constant shaking and constant white light at the same intensity as the seed-culture. When we explored the effect of gene knockdown in the isoprene-producing strains, the cultivation period and concentration of the inducer were optimized using the ISO_crtE strain. The seed-cultures of ISO_EVC, ISO_Hfq and ISO_crtE were cultivated for 2 days in the absence of inducer and an additional day with 0, 2.5 or 5 µM of NiCl_2_. Cultures were prepared using the same concentrations of NiCl_2_ and incubated for 3, 6, 9 and 12 h. Gene knockdown was then addressed for the remaining strains with 0 and 2.5 µM NiCl_2_ and the cultures were incubated for 6 h at 30 °C and constant shaking and constant illumination.

For each time point, 3 tubes were used to assess the performance of the strain. For that, 150 µL of headspace gas were injected in a Clarus 580 (Perkin Elmer) gas chromatograph (GC) equipped with a flame-ionization detector (FID) and a Porapak QS packed column (Porapak QS 80/80 PE 8000, 1.8 m × 2 mm ID, Cat. No. N9305013-ZW5531, Perkin Elmer) and run using the same program as described by Englund et al*.* [18]. The isoprene peak at 1.74 min was compared to a standard curve (isoprene analytical standard, Sigma-Aldrich). Growth was assessed at each time point by measuring the OD_750_ after isoprene quantification and 4 mL of culture from 2 replicates of each strain were sampled, centrifuged and the cell pellets were stored at − 80 °C for posterior RNA extraction. At least two independent experiments were conducted for each case.

### Bisabolene experiments

Cultures of BIS_Hfq, BIS_chlP, BIS_crtB and BIS_sqs were inoculated at OD_750_ = 0.2 into 20 mL BG11 supplemented with 50 mM sodium bicarbonate, 50 mM TES buffer (pH adjusted to 8.0), 25 µg mL^−1^ kanamycin and 10 µg mL^−1^ chloramphenicol. An organic layer was set up by adding 20% (v/v) dodecane (reagent grade, > 99%; Honeywell) to the cultures. Two sets of biological triplicates were grown in simultaneous for 8 days in 100 mL Erlenmeyer flasks at 30 °C and constant fluorescent white light at an intensity of 50 µmol photons m^−2^ s^−1^. Induction of Hfq expression was performed on the second day in one of the sets of cultures by adding NiCl_2_ to a final concentration of 3 µM. The second set of cultures was maintained uninduced. Every 2 days, 2 mL of culture were sampled for growth assessment by OD_750_, and the same volume of new cultivation medium was replenished.

For quantification of the bisabolene present in the organic layer, 200 µL of dodecane layer were sampled every 2 days and transferred to GC vials (VWR) and 200 µL of dodecane were replenished to each culture. The GC vials were stored at − 20 °C until further analysis. The bisabolene content was then assessed via GC-FID (Clarus 580, PerkinElmer), using an Elite-WAX capillary column (30 m × 0.25 mm ID × 0.25 µm film; PerkinElmer). The chromatography conditions were the same as previously described [2] and the (*E*)-α-bisabolene peak at 10.7 min was compared to a standard curve (bisabolene, mixture of isomers, Alfa Aesar).

### Quantitative real-time PCR (RT-qPCR)

The frozen cell pellets were resuspended in 500 µL of TRI Reagent® (DNA, RNA and protein isolation grade, Sigma-Aldrich), 0.2 g of glass beads (acid-washed, Sigma-Aldrich) were added and the samples were then submitted to 3 cycles of vortexing at 5500 rpm for 30 s and incubation on ice for 2 min. The crude extracts were centrifuged at 15,000×*g* at 4 °C for 10 min and the supernatants were then transferred to new microcentrifuge tubes and further processed using the Direct-zol™ RNA Miniprep kit (ZymoResearch). The manufacturer’s recommendations were followed, with exception of the DNase I treatment step, which was performed after the extraction using DNase I (ThemoFisher Scientific). RNA was used for cDNA synthesis using the iScript cDNA synthesis kit (BIO-RAD) and following the protocol from the manufacturer.

RT-qPCR was carried out using the iTaq Universal SYBR Green Supermix (BIO-RAD), in reactions of 10 µL, using 0.3 µM of each primer and 1 µL of 5 × diluted cDNA. Negative controls (no template cDNA) were included and a melting curve analysis was performed in all assays. The PCR was performed using a CFX Connect Real Time PCR system (BIO-RAD). Three technical replicates were performed for each condition. The 2^−ΔΔCT^ method was used to analyse relative expression of each gene, using the *rnpB* gene as reference [51]. The primers used for RT-qPCR are described in Additional file 1: Table S2.

## Supplementary Information

**Additional file 1: Table S1.** DNA sequences used in the assembly of the plasmids harbouring the Hfq-MicC tool. **Table S2.** Primers used for Real-time quantitative PCR (RT-qPCT) and respective annealing temperatures (T_a_).

## Data Availability

The datasets supporting the conclusions of this article are included within the article and its additional files.

## Abbreviations

3PG: 3-Phosphoglycerate
AA: Amino acid
*A. grandis*: *Abies grandis*
ADP: Adenosine diphosphate
AgB: Bisabolene synthase from Abies grandis
asRNA: Antisense RNA
ATP: Adenosine triphosphate
AtpB: β-Subunit of ATP synthase
Bis: (*E*)-α-bisabolene
CBB: Calvin-Benson Bassham
ccmA: 3-Deoxy-d-arabinoheptulosonate 7-phosphate synthase
Chl *a*: Chlorophyll *a*
ChlP: Geranylgeranyl pyrophosphate reductase
CoA: Coenzyme A
CrtB: Phytoene synthase
CrtE: Polyprenyl transferase
DAHP: 3-Deoxy-d-arabinoheptulosonate 7-phosphate
DDH: d-Lactate dehydrogenase
DMAPP: Dimethylallyl pyrophosphate
DXP: Deoxy-d-xylulose 5-phosphate
DXS: Deoxy-d-xylulose-5-phosphate synthase
EgIspS: Isoprene synthase from *Eucalyptus glubulus*
*E. coli*: *Escherichia coli*
*E. globulus*: *Eucalyptus globulus*
E4P: Erythrose 4-phosphate
F6P: Fructose 6-phosphate
FPP: Farnesyl pyrophosphate
G3P: Glyceraldehyde 3-phosphate
G6P: Glucose 6-phosphate
GC: Gas chromatography
GGPP: Geranylgeranyl pyrophosphate
GPP: Geranyl pyrophosphate
HPT: Homogentisate phytyltrasnferase
IDI: Isopentenyl pyrophosphate isomerase
IPP: Isopentenyl pyrophosphate
Iso: Isoprene
Mal: Malate
MEP: Methylerythritol 4-phosphate
OAA: Oxaloacetate
OD: Optical density
PEP: Phosphoenolpyruvate
PEPc: Phosphoenolpyruvate carboxylase
Gpi: Glucose 6-phosphate isomerase
Phytyl-PP: Phytyl pyrophosphate
PP: Pentose phosphate
Pyr: Pyruvate
RT-qPCR: Real-time quantitative PCR
Sqln: Squalene
Sqs: Squalene synthase
*Synechocystis*: *Synechocystis* sp. PCC 6803
TCA: Tricarboxylic acid
ThiG: Thiazole synthase
TPP: Thiamine pyrophosphate
