# Supplementary material for: Mapping competitive pathways to terpenoid biosynthesis in Synechocystis sp. PCC 6803 using an antisense RNA synthetic tool
Source: Microb Cell Fact. 2023 Feb 23;22:35. doi: 10.1186/s12934-023-02040-2 (PMC9951418; doi:10.1186/s12934-023-02040-2)
Supplement: Supplementary file 1 — Additional file 1: Table S1. DNA sequences used in the assembly of the plasmids harbouring the Hfq-MicC tool. Table S2. Primers used for Real-time quantitative PCR (RT-qPCT) and respective annealing temperatures (Ta). [file 12934_2023_2040_MOESM1_ESM.docx]

**Additional file**

**Mapping competitive pathways to terpenoid biosynthesis in *Synechocystis* sp. PCC 6803 using a small RNA synthetic tool**

**Table S1** – DNA sequences used in the assembly of the plasmids harbouring the Hfq-MicC tool.

| **Genetic element** | **DNA sequence (5’ – 3’)** | **Target gene** | **Reference** |
| --- | --- | --- | --- |
| P*_nrsB_* | TCCACCAGCAAAATTCGCATCGCCTCTGCCTTTTTTATAACGGTCTGATCTTAGCGGGGGAAGGAGATTTTCACCTGAATTTCATACCCCCTTTGGCAGACTGGGAAAATCTTGGACAAATTCCCAATT | - | [1] |
| P*_trc1O_* | TTGACAATTAATCATCCGGCTCGTATAATGTGTGGAATTGTGAGCGGATAACAATTTCACACA | - | [2] |
| RBS* | TAGTGGAGGT | - | [3] |
| hfq | ATGGCTAAGGGGCAATCTTTACAAGATCCGTTCCTGAACGCACTGCGTCGGGAACGTGTTCCAGTTTCTATTTATTTGGTGAATGGTATTAAGCTGCAAGGGCAAATCGAGTCTTTTGATCAGTTCGTGATCCTGTTGAAAAACACGGTCAGCCAGATGGTTTACAAGCACGCGATTTCTACTGTTGTCCCGTCTCGCCCGGTTTCTCATCACAGTAACAACGCCGGTGGCGGTACCAGCAGTAACTACCATCATGGTAGCAGCGCGCAGAATACTTCCGCGCAACAGGACAGCGAAGAAACCGAATAA | - | [4] |
| micC | TTTCTGTTGGGCCATTGCATTGCCACTGATTTTCCAACATATAAAAAGACAAGCCCGAACAGTCGTCCGGGCTTTTTTT | - |  |
| TrbcL | GCTGTCGAAGTTGAACATCAGTAAGCAGTGGGTTTTGGCTGATTAACAATTTTTTGACAATTGCCAATGACTAACTGTTAGTTAAGCCGCTGTTAATGGCTAATTAGTTAGCAGACTCAACCCCGAAGAACTGTTCATCCTTAACGGACGAGTACAACTCCGACAATCCAAACACCGGT | - |  |
| asRNA-crtE | AACCATACTGTCACTGGATACTAA | *slr0739* | This study |
| asRNA-ccmA | GCCGACTTTCATGACGACGATCAT | *sll0934* | This study |
| asRNA-crtB | AGACGGTTTGGGCAGTTGTAACAT | *slr1255* | This study |
| asRNA-chlP | AACGACTGCTACCCGTAATACCAA | *sll1091* | This study |
| asRNA-pepC | GAATGCAGGAACTGCCAAGTTCAT | *sll0920* | This study |
| asRNA-ddH | ACTGCTAAAAAAAGCGATTTTCAT | *slr1556* | This study |
| asRNA-pgi | TTGCCAAAGTTGTTGGTTATTCAT | *slr1349* | [5] |
| asRNA-sqs | AAGGGCATTACGACGTAGGCTCAT | *sll0513* | This study |
| asRNA-thiG | GAGAACGTCACTGGTTGTTTGCAT | *slr0633* | This study |
| asRNA-ilvB | GTCTGCGGTGTTCATTTGCCCCAT | *sll1981* | This study |
| asRNA-ilvG | GGGATTAGGAATTGATGCAACCAC | *slr2088* | This study |
| asRNA-atpB | TAGGATGAACAAGGTATTAAGCAT | *sll1324* | This study |

**Table S2 –** Primers used for Real-time quantitative PCR (RT-qPCT) and respective annealing temperatures (T_a_).

| **Name** | **5’ – 3’ sequence** | **Target gene** | **T_a_ (ºC)** | **Reference** |
| --- | --- | --- | --- | --- |
| rnpBF1 | CGTTAGGATAGTGCCACAG | *rnpB** | 56 | [6] |
| rnpBR1 | CGCTCTTACCGCACCTTTG |  |  |  |
| crtE_Fwd | CCTGTGCCCTGGAAATGAT | *crtE* (*slr0739*) | 57 | This study |
| crtE_Rev | CCCGTACACTTTGTGGTTAGT |  |  |  |
| chlP_Fwd | GCTACGGCGCTACCTATTT | *chlP* (*sll1091*) | 56 |  |
| chlP_Rev | CATCGATGTCGGAACACATTTC |  |  |  |
| crtB_Fwd | TGGACAGCGTTGATGTTGTA | *crtB* (*slr1255*) | 56 |  |
| crtB_Rev | CCAGGCCACGGGTAAATATAG |  |  |  |
| sqs_Fwd | ACCAGTCGGACATTCTACATTC | *sqs* (*sll0513*) | 58 |  |
| sqs_Rev | GGGTGGTCTTCCACTTCATC |  |  |  |

***** - *rnpB* was used as reference gene for RT-qPCR analysis.

**References**

1. Englund, E., F. Liang, and P. Lindberg, *Evaluation of promoters and ribosome binding sites for biotechnological applications in the unicellular cyanobacterium Synechocystis sp. PCC 6803.* Sci Rep, 2016. **6**: p. 36640.

2. Huang, H.H., et al., *Design and characterization of molecular tools for a Synthetic Biology approach towards developing cyanobacterial biotechnology.* Nucleic Acids Res, 2010. **38**(8): p. 2577-93.

3. Heidorn, T., et al., *Synthetic biology in cyanobacteria engineering and analyzing novel functions.* Methods Enzymol, 2011. **497**: p. 539-79.

4. Sun, T., et al., *Re-direction of carbon flux to key precursor malonyl-CoA via artificial small RNAs in photosynthetic Synechocystis sp. PCC 6803.* Biotechnol Biofuels, 2018. **11**: p. 26.

5. Wang, X., et al., *Light-Driven Biosynthesis of myo-Inositol Directly From CO2 in Synechocystis sp. PCC 6803.* Front Microbiol, 2020. **11**: p. 566117.

6. Pinto, F., et al., *Selection of Suitable Reference Genes for RT-qPCR Analyses in Cyanobacteria.* PLoS ONE, 2012. **7**(4): p. e34983.
